# Functional Neurological Symptom Disorder Obscured by Incomplete Diagnostic Communication: A Case Report

**DOI:** 10.7759/cureus.115839

**Published:** 2026-09-05

**Authors:** Mark C Jackson, Isabella Ruby, Eleena Akotia, Amirpasha Ehsan

**Affiliations:** 1 College of Osteopathic Medicine of the Pacific, Western University of Health Sciences, Pomona, USA; 2 Rehabilitation, Providence St. Jude Medical Center, Fullerton, USA; 3 Physical Medicine and Rehabilitation, Providence Affiliated Physicians, St. Jude, Fullerton, USA

**Keywords:** care transitions, conversion disorder, diagnostic momentum, fnd, functional neurological symptom disorder, hoovers sign, ptsd, trauma informed care

## Abstract

Functional neurological disorder (FND) is a diagnosis grounded in positive clinical signs, yet it remains vulnerable to underdocumentation and incomplete communication across care transitions, particularly when it is suspected but not formally recorded.

In this case, a 24-year-old woman with a history of major trauma (three gunshot wounds sustained four years earlier) presented with sudden-onset left lower extremity (LLE) weakness, pain, and tingling coinciding with the anniversary month of her injury. Extensive neuroimaging and serologic workup were unrevealing aside from an incidental disc protrusion and elevated angiotensin-converting enzyme (ACE) titers. Due to the presence of localized lower extremity plegia and anesthesia in the absence of structural pathology on neuroimaging, the patient was empirically treated for suspected autoimmune plexopathy with high-dose methylprednisolone, intravenous immunoglobulin (IVIG), and plasmapheresis without sustained benefit. On transfer to a tertiary center, examination revealed fluctuating strength and non-anatomic sensory loss; electromyography was inconclusive. The patient was discharged with a diagnosis of "suspected plexopathy of the lumbosacral plexus," though peer-to-peer consultation later revealed that the tertiary team's leading differential had, in fact, been FND, a suspicion that was never formally documented. On admission to acute inpatient rehabilitation, neuropsychological evaluation suggested a history of post-traumatic stress disorder (PTSD) and a positive Hoover's sign supporting functional weakness. A diagnosis of FND was established, taking into account the entire clinical assessment, including history, physical examination, and multidisciplinary evaluation.

This case demonstrates that a functional diagnosis privately suspected but not formally documented can be effectively lost at a transition of care, exposing the patient to repeated invasive empiric therapy and diagnostic delay. Formally documenting FND as an active differential diagnosis and delivering an early, transparent, biopsychosocially framed explanation to the patient may reduce iatrogenic harm and support engagement in rehabilitation.

## Introduction

Functional neurological disorder (FND), previously known as conversion disorder, is a neuropsychiatric condition in which a person experiences real neurological symptoms that are not fully explained by structural damage or a typical neurological disease process. It affects mainly women and commonly is associated with other psychiatric and neurologic disorders, with a prevalence estimated to be between 79 and 144 per 100,000 people [1]. Symptoms may include weakness, paralysis, tremors, gait disturbance, sensory loss, speech difficulty, visual changes, cognitive symptoms, and seizure-like episodes. Although these symptoms can resemble stroke, epilepsy, multiple sclerosis, or movement disorders, FND is understood as a disorder of nervous system functioning rather than visible injury [2]. The symptoms are involuntary, distressing, and can significantly impair daily functioning.

The pathophysiology of FND is best explained through a biopsychosocial model, in which biological, psychological, and social factors interact. FND has been associated with stress, emotional distress, trauma, anxiety, and environmental triggers, although not every patient has an identifiable stressor. Recent studies have shown that the brain supports our experience through active inference, driving movement and perception through predictions and checking them against sensory input. In FND, symptom-related prior predictions are assigned increased confidence, while actual sensory evidence from the limb is ignored, producing genuinely experienced but self-generating symptoms. Stress can increase these unhelpful expectations and suppress corrective feedback within this system [3]. Further research supports the abnormal communication between brain networks involved in movement, sensation, attention, emotion, stress regulation, and voluntary control. Physiological findings include altered limbic system activity and abnormal connectivity between emotional-processing regions and motor-control networks, which may explain symptom fluctuation, worsening with stress or attention, and improvement with distraction [4].

FND is diagnosed through clinical history, neurological examination, and positive signs such as Hoover's sign, tremor entrainment, symptom variability, and inconsistency between observed and tested function [5]. Treatment often involves neurology, psychology or psychiatry, physical therapy, occupational therapy, speech therapy, and primary care. Although FND is common in neurology settings, it is often underdiagnosed or misdiagnosed due to diagnostic uncertainty, overlap in symptoms with other neurologic diseases, variance in clinician experience, and possibly stigmatization [6].

This case examines FND and the importance of interpreting neurological symptoms within the larger clinical picture. Approaching FND as a rule-in diagnosis rather than as a rule-out diagnosis when suspected may help clinicians recognize similar cases earlier, guiding treatment, expediting early multidisciplinary care, and avoiding communication failures.

## Case presentation

History and presentation

In April 2026, a 24-year-old female with a history of depression and anxiety managed with alprazolam presented with a two-day history of left lower extremity (LLE) weakness, pain, and tingling. Her medical history was notable for three gunshot wounds to the left lower abdomen, left flank, and right upper leg, sustained in a drive-by shooting four years prior. Her current presentation directly coincided with the anniversary month of this trauma.

Initial diagnostic workup and empirical treatment

Initial magnetic resonance imaging (MRI) of the brain and entire spine revealed only a minimal L5-S1 disc protrusion (Figures 1-2). Computed tomography (CT) of the abdomen and pelvis showed no structural mass (Figure 3). Serum workup, including HIV, ANA, ANCA, RA, SSA, SSB, Lyme, and heavy metals, was unremarkable, though serum angiotensin-converting enzyme (ACE) titers were elevated at 95 (reference range, 14-28). The elevated ACE level was determined to be insignificant due to being nonspecific in isolation; a chest radiograph to rule out sarcoidosis was normal (Figure 4). Renal artery imaging to rule out renal artery stenosis was normal.

**Figure 1:**
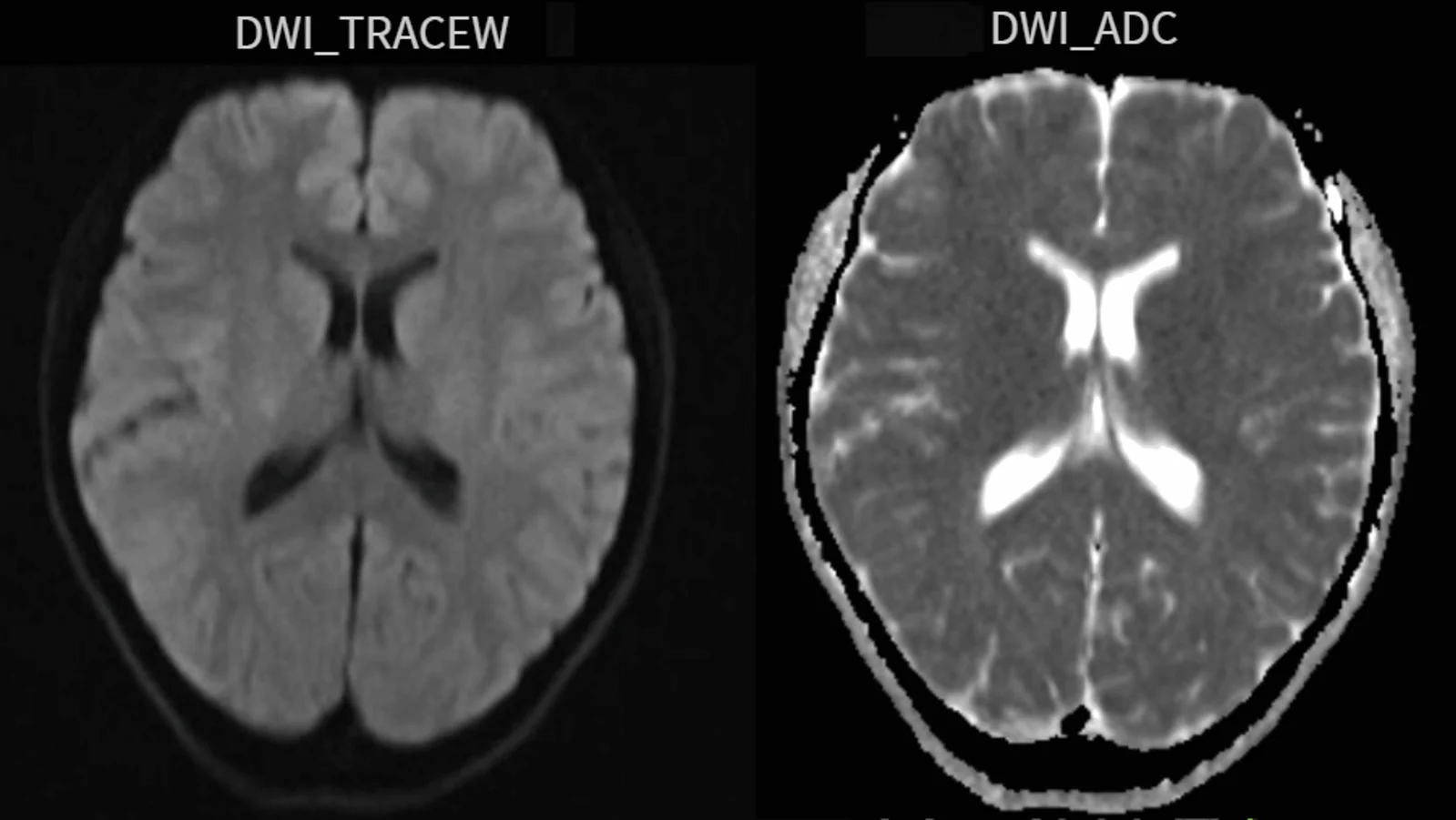
Axial DWI and ADC MRI of the Brain MRI brain showing no evidence of acute infarct and no evidence of acute intracranial abnormalities. DWI: diffusion-weighted imaging; ADC: apparent diffusion coefficient; MRI: magnetic resonance imaging

**Figure 2:**
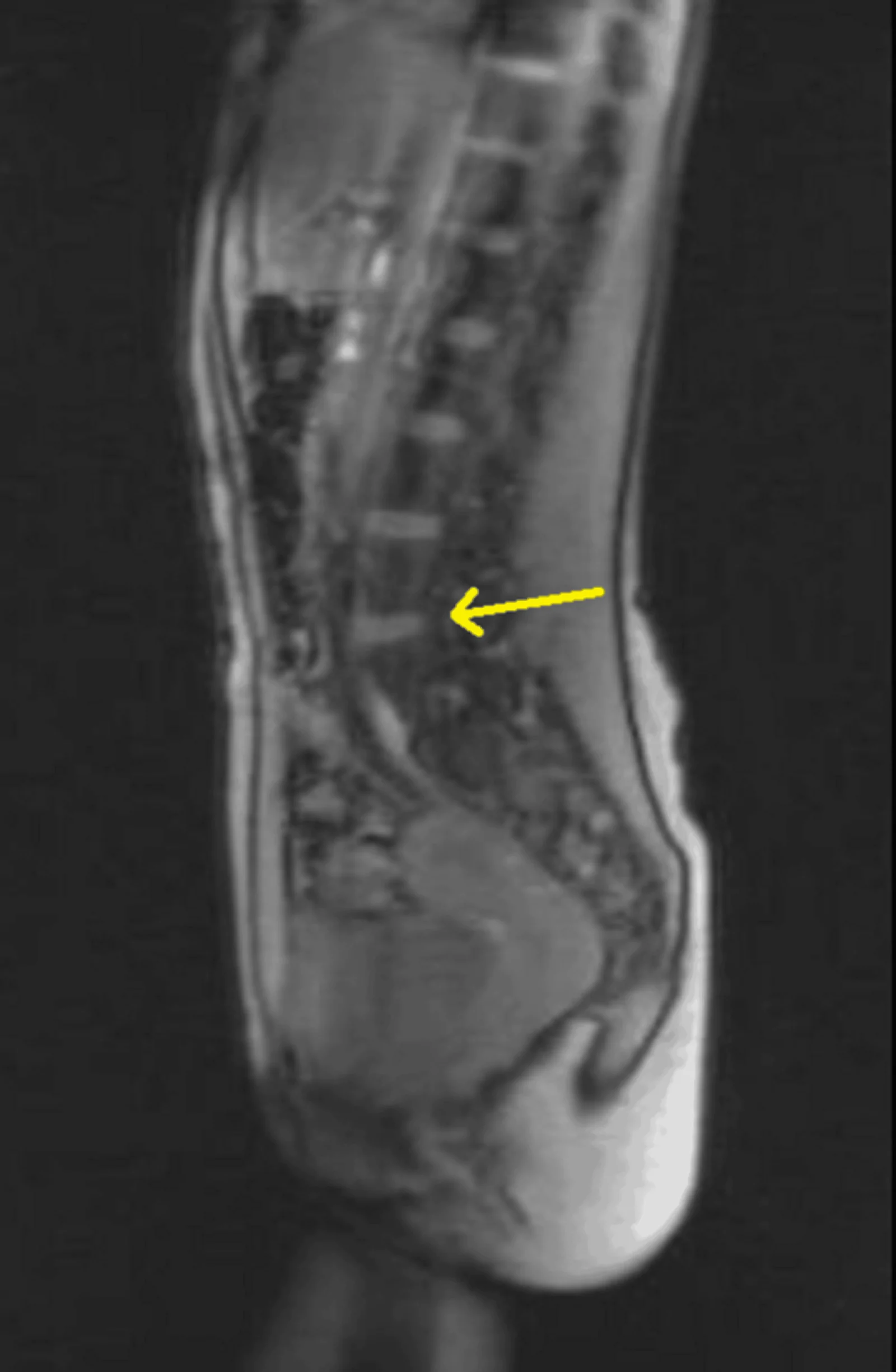
Sagittal MRI of the Lumbar Spine Sagittal MRI of the lumbar spine showing a minimal L5-S1 posterior disc protrusion, indicated by the yellow arrow; otherwise, it is unremarkable. MRI: magnetic resonance imaging

**Figure 3:**
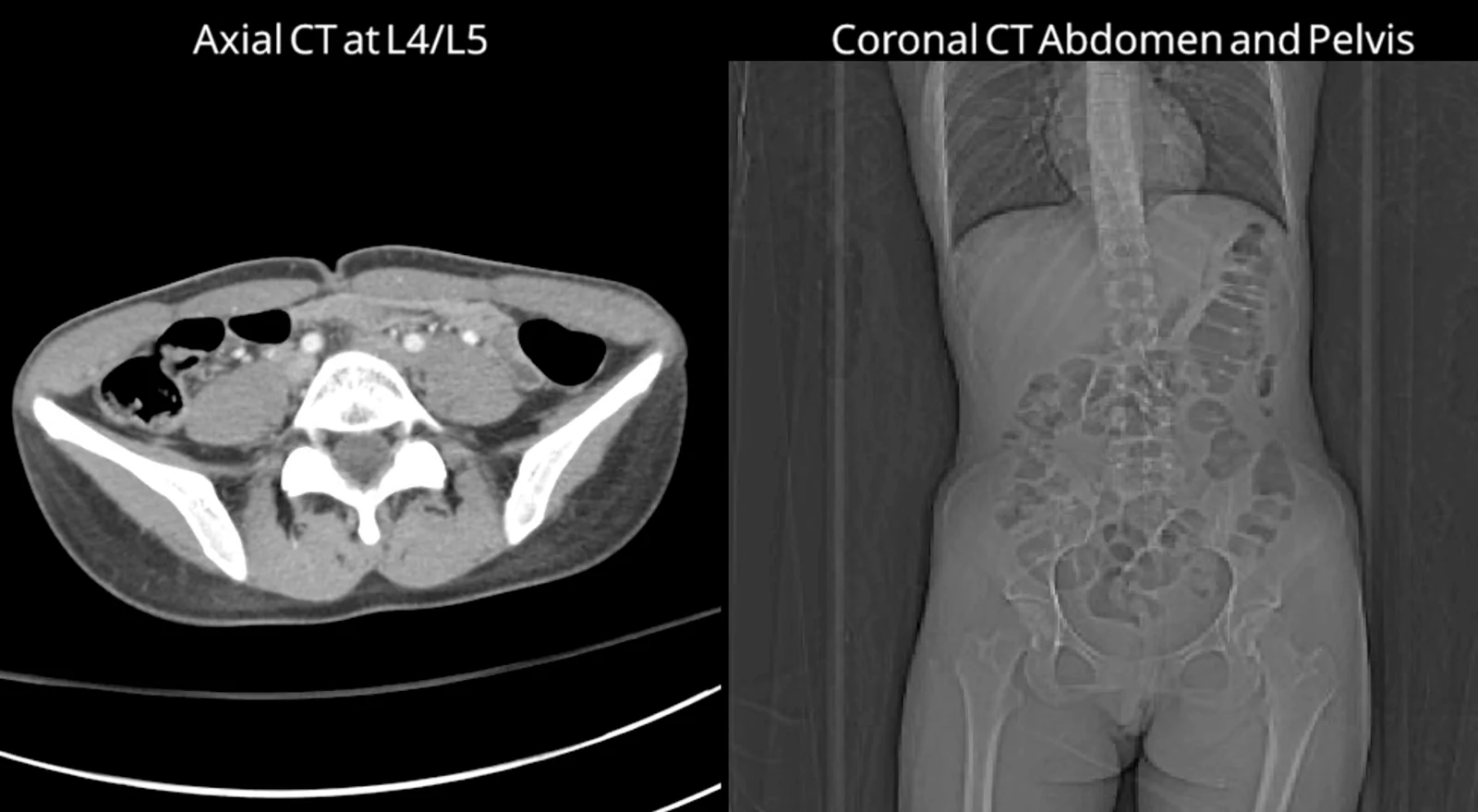
CT of the Abdomen and Pelvis CT of the abdomen and pelvis with no evidence of structural abnormality. CT: computed tomography

**Figure 4:**
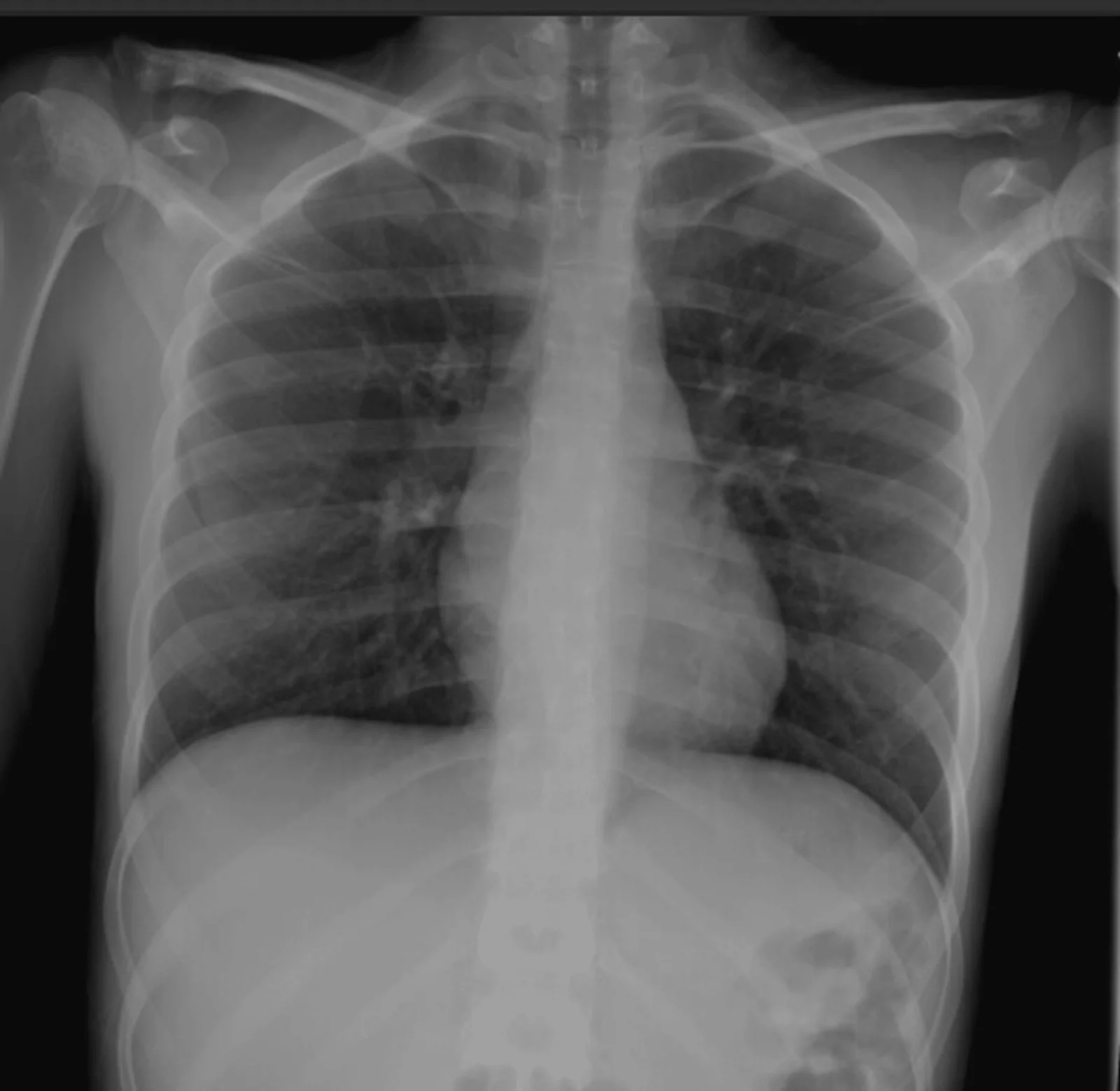
Anterior Posterior Chest Radiograph Anteroposterior chest radiograph demonstrating clear lungs without focal consolidation, pleural effusion, or pneumothorax.

The patient was suspected to have autoimmune plexopathy. Empiric treatment with high-dose methylprednisolone produced initial, transient improvement in left leg movement, followed by subsequent worsening weakness and loss of sensation to the left hip. The patient was further treated with five days of intravenous immunoglobulin (IVIG) and six days of plasmapheresis without clinical response.

Physical examination and electrodiagnostics

Upon transfer to a tertiary center for further diagnostic workup, cranial nerves II-XII were intact. On motor examination, the right lower extremity and bilateral upper extremities demonstrated full muscle strength, while the LLE demonstrated variable/impaired strength across movements (Table 1). Sensory examination revealed absolute anesthesia to touch, pinprick, and temperature below the left knee, with fluctuating sensation symptoms medially and laterally above the knee and below the left hip, with otherwise intact sensation. Deep tendon reflexes were symmetrical and brisk at the patella and Achilles bilaterally, with absent Babinski and Hoffman signs. Electromyography (without nerve conduction study) of the LLE was unrevealing, demonstrating only a lack of muscle activation in the LLE. The patient was discharged with suspected plexopathy of the lumbosacral plexus due to the acute onset of LLE weakness and sensory deficit.

**Table 1:** Summary of Motor Strength Examination Findings (Medical Research Council Muscle Strength Scale Ratings)

Anatomical Region/Movement Tested	Motor Strength Rating
Bilateral Upper Extremities	5/5
Right Lower Extremity (RLE)	5/5
Left Lower Extremity (LLE) - Hip Flexion	3/5
Left Lower Extremity (LLE) - Knee Flexion/Extension	2/5
Left Lower Extremity (LLE) - Dorsiflexion	0/5
Left Lower Extremity (LLE) - Plantar Flexion	0/5

Rehabilitation and hospital course

Due to ongoing impairments in mobility and activities of daily living, the patient was admitted to acute inpatient rehabilitation. Neuropsychological evaluation, including clinical interview and the Post-Traumatic Stress Disorder Checklist for DSM-5 (PCL-5), revealed a trauma history extending beyond the gunshot injury and a PCL-5 score of 67/80 spanning all four DSM-5 symptom clusters, supporting a provisional diagnosis of severe post-traumatic stress disorder (PTSD) [7]. Neuropsychological session content and the patient's clinical response are summarized in Table 2. Given her history of psychological stress and the absence of neurological findings on diagnostic studies, FND was considered. A positive Hoover's sign was demonstrated, supporting functional weakness of the LLE in the context of the entire clinical assessment. Peer-to-peer consultation with the tertiary institution subsequently revealed that the primary team had suspected FND as their leading differential, but this diagnosis had not been formally documented or clearly communicated at the time of transfer of care.

**Table 2:** Neuropsychological Evaluation and Session Summary

Session	Timing	Focus/Intervention	Documented Response
Initial evaluation	Admission to rehabilitation	Clinical interview and PCL-5 administration; emotional processing of trauma history; psychoeducation on trauma, anxiety, and nervous system regulation; exploration of somatic reactions during the anniversary month; reinforcement of adaptive coping strategies	PCL-5 score 67/80, with symptoms across all four DSM-5 post-traumatic stress disorder (PTSD) symptom clusters (intrusion, avoidance, negative alterations in cognition/mood, arousal/reactivity)
Follow-up session	~1 week later	Continued emotional processing; review of progress in outpatient psychotherapy	Improved mood; denial of significant depressive/anxiety symptoms

## Discussion

Documentation omission

This case highlights communication pitfalls surrounding the diagnosis and management of FND. The tertiary team was contacted by the treating team on the acute inpatient rehabilitation unit to further clarify clinical reasoning and documentation. The treating provider admitted to suspecting FND as their top differential; however, their formal discharge documentation omitted this suspicion entirely, offering only a vague note: "Query functional symptoms given many previous trauma." The disconnect between the tertiary center's internal clinical suspicion and the information explicitly communicated to the patient and the acute inpatient rehabilitation unit could indicate bias, in which providers fail to detect or document a psychological or functional diagnosis. In an urban HIV population, rates of specific diagnoses such as PTSD and adjustment disorder were markedly lower than national estimates, while major depression was documented at expected rates, suggesting the presence of selective detection or documentation bias [8]. Further, in studies with German general physicians and psychiatrists, fear of patient stigmatization and deliberate coding of an alternative somatic diagnosis were identified as explicit motives for withholding psychiatric diagnostic codes [9]. Combined evidence of selective detection or documentation biases in populations with similar psychiatric diagnoses supports that a similar pattern may exist for documentation of FND. Future research should be done to further evaluate this.

In this case, the patient was transitioned to the rehabilitation unit with a primary diagnosis of "suspected plexopathy of the lumbosacral plexus," which had already failed to respond to high-dose methylprednisolone, IVIG, and six days of plasmapheresis. By failing to document this suspicion in the medical record, the transferring facility inadvertently fragmented continuity of care, forcing the rehabilitation team to independently rediscover the diagnostic clues.

On rehabilitation admission, the team shifted the differential to FND due to the patient's positive clinical signs, unrevealing diagnostics, and psychosocial history. Normal imaging, inconclusive electrodiagnostics, and the failure of aggressive immunomodulatory therapies made autoimmune plexopathy unlikely. The team considered FND due to the patient's fluctuating motor strength and non-anatomic sensory loss. A positive Hoover's sign further pointed to a functional cause of neurological symptoms. Contextualizing these neurological findings with her psychiatric history, including symptom onset coinciding with the anniversary of her gunshot wounds, indicated a biopsychosocial etiology. This integrated assessment caused the rehabilitation team to establish FND as the leading diagnosis and pivot to targeted, trauma-focused multidisciplinary rehabilitation.

Stigma of psychiatric diagnoses and why FND is particularly exposed

More broadly, reluctance to document this suspicion reflects a pattern that extends beyond this case. Diagnoses with a psychological basis are more subjectively diagnosed than those with a demonstrable structural, biochemical, or radiographic correlation that can be measured [6]. Further, clinicians may avoid recording such an impression out of concern that a psychiatric label will be read by the patient as dismissive, that it will inappropriately follow the patient into unrelated future encounters, or simply out of uncertainty about how to frame a diagnosis that falls outside their own specialty's traditional scope [9]. The effect is that a clinically important hypothesis can disappear from the record entirely, leaving only an unconfirmed diagnostic label to anchor subsequent care.

FND is especially exposed to this pattern, as it sits at the interface of neurology and psychiatry. It was also historically equated with fabrication or weakness of character, despite FND symptoms being involuntary and despite the diagnosis increasingly resting on positive examination findings, such as Hoover's sign, rather than on exclusion alone [6]. Finally, because FND was previously defined as a diagnosis of exclusion, clinicians may feel obligated to exhaust the structural or physical-organic differential before raising it, even when positive functional signs are evident much earlier [5]. Together, these factors make FND a diagnosis particularly vulnerable to incomplete documentation and communication.

Diagnostic momentum

The primary danger of omitting a suspected FND diagnosis while documenting an unconfirmed organic label such as "suspected lumbosacral plexopathy" is the future risk of diagnostic momentum - a systemic cognitive bias in which a preliminary diagnostic hypothesis gathers uncontested acceptance as it is passed along a patient's longitudinal care timeline [10]. When an institution discharges a patient with an unresolved organic diagnosis, that label becomes embedded in the electronic health record as an established clinical baseline. Should this patient present to a different emergency department or neurology clinic during a future anniversary month or period of high stress, subsequent clinicians will naturally anchor to the documented history of "plexopathy" [10,11].

Because clinicians inherently trust prior specialized workups, the tentative organic label acts as an anchor that is highly likely to trigger a new wave of diagnostic momentum [11]. This may delay consideration of a functional etiology and contribute to repetitive re-evaluation that may not ultimately alter management, thereby postponing appropriate diagnosis and treatment of conditions such as FND. This delay in care is not benign; it actively exposes the patient to severe, preventable iatrogenic risks through the escalation of empirical therapies [12]. In this case, empiric treatment of autoimmune plexopathy was continued while suspected. One empiric treatment in this case was high-dose methylprednisolone. While no adverse effects were recorded in this case, methylprednisolone carries a risk of unwanted side effects [13].

The prolonged diagnostic delay associated with diagnostic momentum may also carry important psychological consequences. Prolonged diagnostic uncertainty, particularly when accompanied by invasive treatments that fail to produce meaningful improvement, may contribute to patient distress, frustration, and degradation of trust in the healthcare system [14]. In addition to delaying appropriate treatment, repeated evaluations for an unconfirmed organic diagnosis may reinforce illness uncertainty and complicate future clinical encounters. Emerging evidence suggests that early recognition and explanation of FND may improve patient engagement, reduce unnecessary diagnostic testing, and facilitate earlier participation in targeted rehabilitation [12,14]. Contemporary approaches emphasize the use of positive clinical signs, such as Hoover's sign, rather than framing FND exclusively as a diagnosis of exclusion [5]. Delayed recognition may prolong disability, increase healthcare utilization, and expose patients to unnecessary procedures or treatments [12]. These findings further underscore the importance of documenting and communicating functional diagnostic considerations as soon as they become clinically relevant.

Communication in transfer

This case demonstrates a critical failure point at the interface between tertiary care and rehabilitation: the gap between what clinicians privately suspect and what they formally communicate [15]. During peer-to-peer consultation, the tertiary team disclosed that FND was their leading differential, yet the discharge summary carried only "suspected plexopathy of the lumbosacral plexus" as the working diagnosis, with the functional possibility reduced to a single equivocal line. This created an asymmetry between verbal and written handoff: information available informally to one clinician was not embedded in the record that would travel with the patient.

The consequences of this asymmetry were immediate. The rehabilitation team began their evaluation anchored to an organic diagnosis that had already failed three escalating immunomodulatory interventions and had to independently reconstruct the diagnostic reasoning the tertiary team had already worked through. Only a dedicated neuropsychological evaluation and elicitation of Hoover's sign allowed the diagnosis to surface. Had these steps not occurred or occurred later, the patient may have been managed indefinitely under the plexopathy framework, with renewed organic workup at any future transition point.

This pattern reflects a broader structural problem in how diagnostic uncertainty is transmitted across care settings. Verbal peer-to-peer exchanges, while valuable for real-time decision-making, are not retrievable by downstream teams and create no durable record [15]. When a functional or psychiatric differential is voiced only in this informal channel, its persistence depends entirely on individual memory and is lost the moment the patient changes institutions, providers, or shifts [15]. Effective transfer communication for suspected FND requires that the differential be documented with the same weight as organic possibilities, not relegated to a hedge phrase that can be overlooked or that fails to prompt further evaluation.

Communication to the patient

Equally significant is what was, and was not, communicated to the patient herself. Over the course of her workup, this patient underwent high-dose corticosteroids, five days of IVIG, and six days of plasmapheresis, each carrying meaningful risk and burden, while functional weakness was suspected internally without resolution. From the patient's perspective, she experienced an escalating sequence of invasive treatments that produced no improvement, with no clear explanation offered for why these interventions were not working or what alternative explanations were being considered. While uncertainty is an inherent part of clinical practice, the principal communication issue in this case was the initial withholding of the leading differential diagnosis.

This matters because how and when a functional diagnosis is communicated substantially affects patient acceptance and outcome [2,5,14]. When patients perceive that a diagnosis is being withheld, hedged, or only reluctantly offered after every organic possibility has been exhausted, FND can be experienced as a diagnosis of exhaustion or last resort. Even though FND was the leading differential of the managing team, this framing risks patients hearing the diagnosis as invalidating, particularly after they have already undergone treatments premised on an organic cause [14]. Multiple studies have demonstrated that patient acceptance of FND is strongly influenced by how the diagnosis is presented. Explanations emphasizing abnormal nervous system functioning, rather than the absence of disease, are associated with greater understanding and engagement in treatment. Conversely, when the diagnosis is presented only after extensive negative testing or framed as one reached after all other possibilities have been exhausted, patients may perceive it as dismissive [2,5]. This case illustrates how early, transparent communication may help patients develop a coherent understanding of their symptoms while facilitating appropriate treatment.

In this case, the diagnosis was ultimately delivered in the context of trauma-focused therapy addressing PTSD related to the patient's prior shooting, with the temporal link to the anniversary month providing a coherent explanatory framework. This integrated approach - presenting the functional symptoms as a real and explainable manifestation of the nervous system's response to trauma and stress, rather than as the absence of organic disease - offers a model for how the diagnosis can be framed constructively.

Strategies to improve transitions of care in suspected FND

Several strategies, informed by the gaps this case exposes, may improve transitions of care when FND is suspected:

Documentation of suspected functional diagnoses as active differentials could increase communication between treatment teams: When FND is raised during clinical discussion, including informal peer-to-peer consultation, entering the diagnosis into the formal record with the same structure given to organic differentials, including supporting findings such as a positive Hoover's sign, symptom variability, or inconsistency between observed and tested function, could better communicate the provider's clinical assessment [8,9,11].

Framing the diagnosis through a biopsychosocial and neuroscience-based model when communicating with patients could increase acceptance: Linking functional symptoms to a real, measurable interaction between the nervous system, stress, and trauma - particularly when a temporal trigger such as an anniversary date is identifiable - may increase acceptance and engagement with treatment [2,5,14]. Early introduction of this framing, as soon as functional features are recognized, could ease patient uncertainty rather than waiting until other explanations have failed.

Ensuring early multidisciplinary involvement could improve outcomes: Including psychology or psychiatry at the point functional features are first noted, rather than only after rehabilitation admission, increases the level and duration of care for patients [12,14]. Earlier involvement of neuropsychological evaluation in this case might have identified the PTSD-FND link and the relevant anniversary timing before three separate high-risk empirical therapies were administered.

Limitations

This report has several limitations. As a single-patient case report, conclusions regarding communication practices and diagnostic decision-making cannot be generalized broadly. Omission of a nerve conduction study limits the evaluation of peripheral neuropathy and plexopathy and represents a diagnostic limitation. Similarly, the psychiatric assessment relied on a single self-report measure, the PCL-5, and a clinical interview rather than repeat standardized outcome measurements; clinical improvement during rehabilitation was documented narratively (patient-reported mood improvement and denial of significant depressive or anxiety symptoms) rather than quantitatively, limiting the ability to establish a relationship between the psychological intervention and symptom resolution. Additionally, direct insight into the tertiary team's rationale for omitting FND from formal documentation was unavailable and was further complicated by a lack of direct communication with the treating neurologists. It is possible that diagnostic uncertainty, rather than reluctance to document a functional diagnosis, contributed to the wording of the discharge summary. In addition, this manuscript is unable to establish a causal relationship between incomplete communication and the patient's clinical outcomes. Nevertheless, the case demonstrates how incomplete communication of diagnostic reasoning can influence downstream clinical care and contribute to diagnostic delay.

## Conclusions

This case highlights that a diagnosis of FND can be lost to communication and documentation gaps during transitions of care. Because the tertiary team's suspicion for FND was never formally communicated or documented, the patient received empiric treatment for a plexopathy when FND was the tertiary team's top diagnosis. This exposed the patient to potential iatrogenic risk and delayed diagnosis. The diagnosis surfaced only after a dedicated neuropsychological evaluation and elicitation of Hoover's sign while in inpatient rehabilitation.

Two lessons can be taken away: functional differentials and the supporting positive findings that justify them should be documented with the same rigor as other diagnoses when suspected. Secondly, screening for psychiatric and functional signs earlier in the workup, rather than after an organic differential is exhausted, may reduce both diagnostic delay and potential iatrogenic harm.
